# Engineered Interfacial Control for Suppression of Phase Instability: Operando Visualization from Device to Module Scale

**DOI:** 10.1002/advs.74909

**Published:** 2026-04-02

**Authors:** Hangyeol Choi, Soo Woong Jeon, Wookyung Jeon, You‐Hyun Seo, Gill Sang Han, Myunghun Shin, Ji‐Sang Park, Jun Hong Noh, Yohan Yoon

**Affiliations:** ^1^ Department of Materials Engineering Korea Aerospace University Goyang Republic of Korea; ^2^ School of Civil, Environmental and Architectural Engineering Korea University Seoul Republic of Korea; ^3^ Division of Advanced Materials Korea Research Institute of Chemical Technology (KRICT) Daejeon Republic of Korea; ^4^ School of Electronics and Information Engineering Korea Aerospace University Goyang Republic of Korea; ^5^ SKKU Advanced Institute of Nano Technology Sungkyunkwan University Suwon Republic of Korea

**Keywords:** deep‐level transient spectroscopy (DLTS), halide phase dynamics, mixed‐halide perovskites, operando visualization, real‐time current density–voltage absorption spectroscopy (RTJAS)

## Abstract

Phase instability in wide‐bandgap mixed‐halide perovskites remains a critical challenge for achieving long‐term stable perovskite solar cells. Here, real‐time current density–voltage absorption spectroscopy (RTJAS) with a transparent indium tin oxide top contact enables operando, spatially resolved visualization of phase evolution across charge‐extracting and charge‐non‐extracting regions under operational bias. Light‐induced phase separation preferentially initiates in regions with locally insufficient carrier extraction, revealing spatial heterogeneity in phase stability. By modifying the interfacial configuration, device‐level RTJAS measurements under identical operating conditions exhibit different phase evolution behaviors, motivating defect level analysis. Deep‐level transient spectroscopy (DLTS) and density functional theory (DFT) modeling indicate that this contrast is associated with differences in interfacial defect landscapes and vacancy‐related energetics. Importantly, operando RTJAS mapping at the module scale reveals that electrically inactive dead areas intrinsic to monolithic interconnect architectures act as preferential sites for the onset of phase instability. Together, these results establish that phase instability in mixed‐halide perovskites is governed by localized charge extraction conditions and interfacial defect energetics. The spatially resolved operando framework presented here provides a diagnostic tool for identifying latent degradation pathways and offers design guidelines for achieving stable and scalable perovskite photovoltaics from device to module scale.

## Graphical abstract

Operando real‐time current density–voltage absorption spectroscopy visualizes spatial phase evolution in mixed‐halide perovskites from device to module scale. Phase instability preferentially initiates in regions with insufficient carrier extraction, revealing electrical boundary conditions as governing factors. Interfacial defect engineering suppresses vacancy‐mediated segregation, providing a scalable strategy for enhancing operational stability.

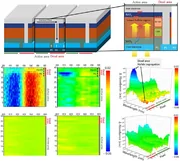

## 1 Introduction

Wide‐bandgap mixed‐halide perovskites, such as CsPbI_2_Br, are promising materials for the fabrication of efficient, stable solar cells owing to their high thermal stabilities and tunable optoelectronic properties [1, 2, 3, 4]. However, these materials are unstable under illumination and electrical bias primarily due to halide segregation [5, 6], which involves the spontaneous redistribution of iodide and bromide ions into phase‐separated regions to form iodine‐rich domains with lower band gaps. These domains act as nonradiative recombination centers and reduce both the open‐circuit voltage and the long‐term device stability [5, 7].

Halide segregation is associated with ionic defect dynamics, particularly the migration of iodine vacancies (V_I_), which act as halide transport pathways under operational conditions [8, 9]. As segregation progresses, these mobile defects can transform into electrically active deep trap states that degrade the photovoltaic (PV) performance. Several strategies have been developed to mitigate segregation by limiting V_I_ formation and mobility, including interface engineering and defect passivation [10, 11, 12]. However, some key questions regarding the initiation of segregation and its correlation with interfacial fields and defects remain unanswered [7, 11, 13].

These challenges are exacerbated in large‐area or monolithic devices, which inherently contain laterally heterogeneous charge‐non‐extracting regions (commonly referred to as “dead areas”) that are electrically connected but do not contribute to power generation [14]. Upon illumination, these regions cannot extract photogenerated charge carriers, resulting in recombination losses, local electrical field reconstruction, and ionic redistribution [15]. Such regions are unavoidable in practical modules and may induce or accelerate halide dissociation. However, the segregation mechanisms in these regions are poorly understood owing to a lack of spatially resolved operando measurements.

To address these limitations, this study integrates real‐time current density–voltage (*J–V*) absorption spectroscopy (RTJAS) [10, 16], deep‐level transient spectroscopy (DLTS), and density functional theory (DFT) modeling to investigate halide segregation and its suppression in CsPbI_2_Br perovskite solar cells (PSCs) and modules. RTJAS simultaneously captures the spatially resolved absorbance and electrical output during device operation, providing a direct view of phase segregation evolution across both charge‐extracting and charge‐non‐extracting regions. DLTS quantifies the trap states and activation energies associated with V_I_ [17], while DFT modeling reveals the energetics of V_I_ formation, migration, and interfacial stabilization. This approach demonstrates that halide segregation preferentially initiates in charge‐non‐extracting regions, even under maximum power point tracking (MPPT) conditions, and later propagates to charge‐extracting zones via lateral ion transport. The role of poly(3‐hexylthiophene) (P3HT) in immobilizing V_I_ is evaluated, and its mechanism of suppressing ion migration is examined. This study links ion migration, phase segregation, and defect formation, highlighting interfacial defect engineering as a viable strategy to enhance the intrinsic stabilities of mixed‐halide PSCs.

## 2 Results and Discussion

### 2.1 Operando Identification of the Spatial Origin of Phase Instability

**FIGURE 1:**
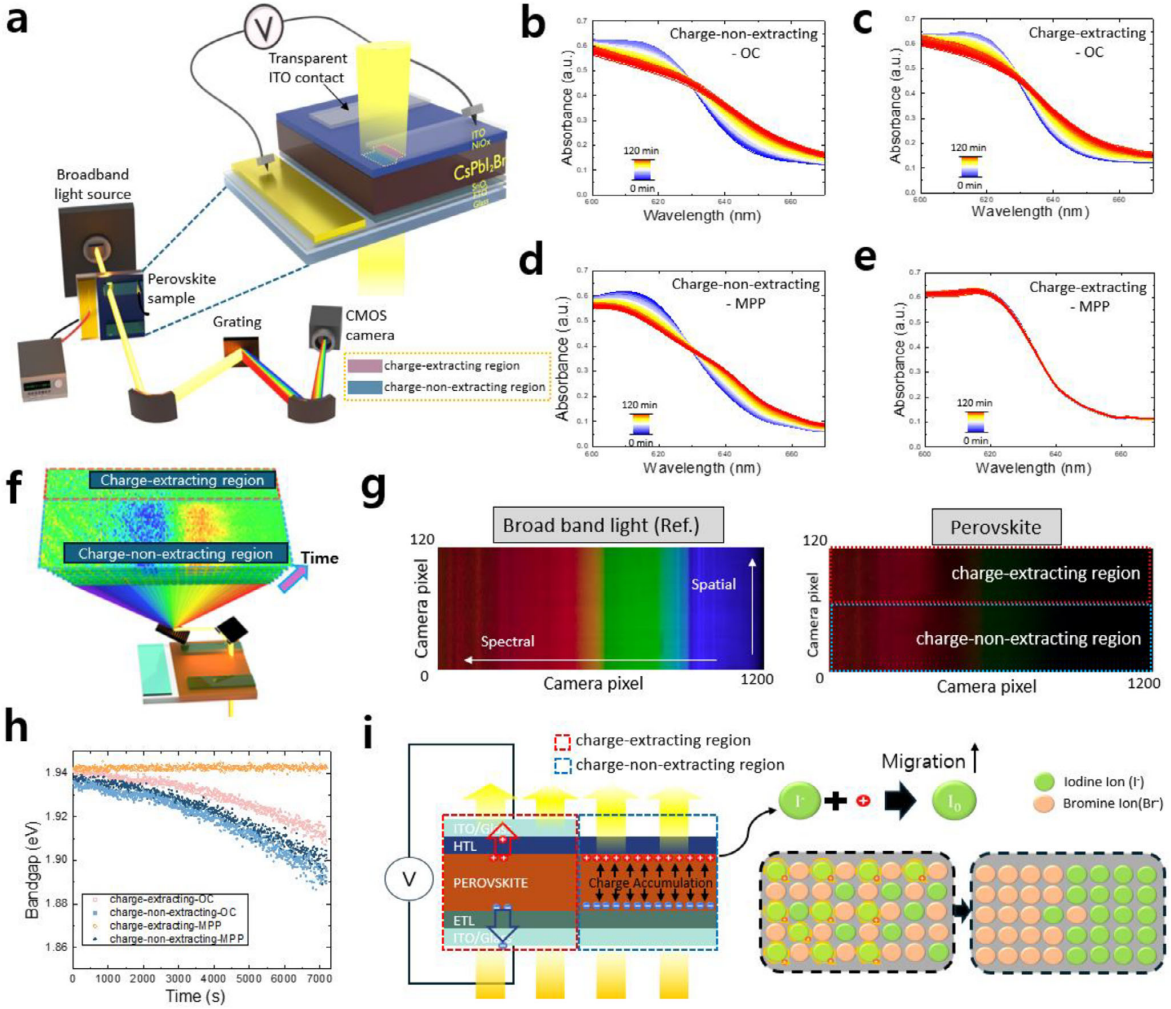
(a) Schematic representation of the RTJAS setup, enabling simultaneous optical measurements in both the charge‐extracting and charge‐non‐extracting regions using a top contact composed of transparent ITO. (b, c) Time‐resolved absorbance spectra under OC conditions in the (b) charge‐non‐extracting and (c) charge‐extracting regions. (d, e) Absorbance spectra recorded under MPP conditions in the (d) charge‐non‐extracting and (e) charge‐extracting regions. (f) Detailed schematic showing the generation of a hyperspectral 2D absorbance map from the complementary metal‐oxide semiconductor (CMOS) sensor, highlighting spectral evolution across both the charge‐extracting and charge‐non‐extracting regions over time. (g) Raw spectral images captured by the CMOS camera: Reference image of broadband light without the sample (left) and the transmission spectrum of the illuminated perovskite device (right), showing separation between the charge‐extracting and charge‐non‐extracting regions. (h) Time‐dependent bandgap evolution extracted from the absorption spectra under different operating conditions. (i) Schematic representation of the charge accumulation and halide segregation mechanisms, showing the oxidation of I^−^ to I^0^ and subsequent segregation in the presence of accumulated holes.

Figure 1a illustrates the RTJAS platform used to simultaneously monitor the optical absorption and *J–V* characteristics across charge‐extracting and non‐extracting regions within the same perovskite device. The platform design, detailed in our previous report [10], employs a transparent indium tin oxide (ITO) top contact to allow localized carrier extraction while preserving optical access to the absorber. This enables spatially resolved analysis of light‐induced spectral evolution under uniform illumination and operational bias. As shown in the raw hyperspectral image (Figure 1g), the absorbance is resolved both spectrally and laterally, allowing real‐time mapping of compositional changes with full spatiotemporal fidelity. This configuration eliminates sample‐to‐sample variation and directly correlates local carrier extraction conditions with phase evolution.

The uniqueness of RTJAS lies in its ability to continuously capture spectral signatures associated with phase evolution across distinct regions of a working device. By comparing optical and electrical responses in regions with and without carrier extraction within the same device, this method enables direct assessment of how local extraction conditions influence light‐induced compositional and structural changes during operation [18, 19]. Consequently, the optical and electrical responses in the regions with and without carrier extraction within the same device can be compared. This method also eliminates sample‐to‐sample variability and enables direct assessment of the effects of charge extraction on light‐induced compositional and structural changes during device operation.

Notably, the spectral changes under illumination differed between the charge‐extracting and non‐extracting regions. Under open‐circuit (OC) conditions, in which photogenerated carriers are not extracted but instead accumulate and recombine [20], both the charge‐extracting and charge‐non‐extracting regions exhibited progressive red‐shifts in their absorption edges from ∼620 to 640 nm over 120 min (Figure 1b,c) [6]. The red‐shift indicates a reduction in the band gap of the absorber layer, consistent with previous reports linking light‐induced halide segregation to the formation of iodine‐rich domains with smaller band gaps [21, 22]. In addition to the redshift of the absorption onset associated with iodine‐rich domains, a subtle but systematic evolution of absorbance is also observed around 590 nm (Figure S1), consistent with redistribution toward bromine‐enriched regions during segregation. The comparable red‐shift in both regions under OC conditions indicates that halide segregation is driven by excess carrier accumulation or a weakened internal electric field.

Under maximum power point (MPP) operation, the phase evolution behavior diverged between the two regions. The charge‐non‐extracting region exhibited a red‐shift and an enhanced long‐wavelength absorbance (Figure 1d), while the charge‐extracting region showed minimal spectral changes (Figure 1e). As confirmed by the bandgap evolution (Figure 1h), light‐induced phase separation is strongly correlated with the absence of efficient carrier extraction. These observations indicate that local carrier dynamics play a decisive role in determining the spatial heterogeneity of phase evolution under operational conditions [20, 23, 24].

Although the absorption data do not directly identify the migrating species, the observed red‐shift is consistent with the formation of iodine‐rich, lower‐bandgap regions. As illustrated in Figure 1i, carrier accumulation in charge‐non‐extracting regions can promote the oxidation of halide ions into neutral halogens under continuous illumination [25, 26]. This process is more favorable for iodine, which is photochemically less stable than bromine [27]. The resulting I^0^ species, which are smaller and more mobile than I^−^, may enhance halide diffusion and facilitate the growth of iodine‐rich regions [25, 28] while depleting the original mixed‐halide regions. The charge accumulation‐driven bandgap shift is shown in Figure S2. The proposed mechanism aligns with previous reports on photoinduced oxidation/redox reactions in perovskites and will be further examined through defect spectroscopy and DFT calculations. Importantly, this behavior is not limited to charge‐non‐extracting regions. Under OC conditions, suppressed charge extraction may lead to similar photocarrier accumulation across the device, rendering even charge‐extracting regions susceptible to phase segregation. Under MPP conditions, the minimal change in absorption in the charge‐extracting region highlights the essential role of carrier dynamics in maintaining phase stability during illumination.

**FIGURE 2:**
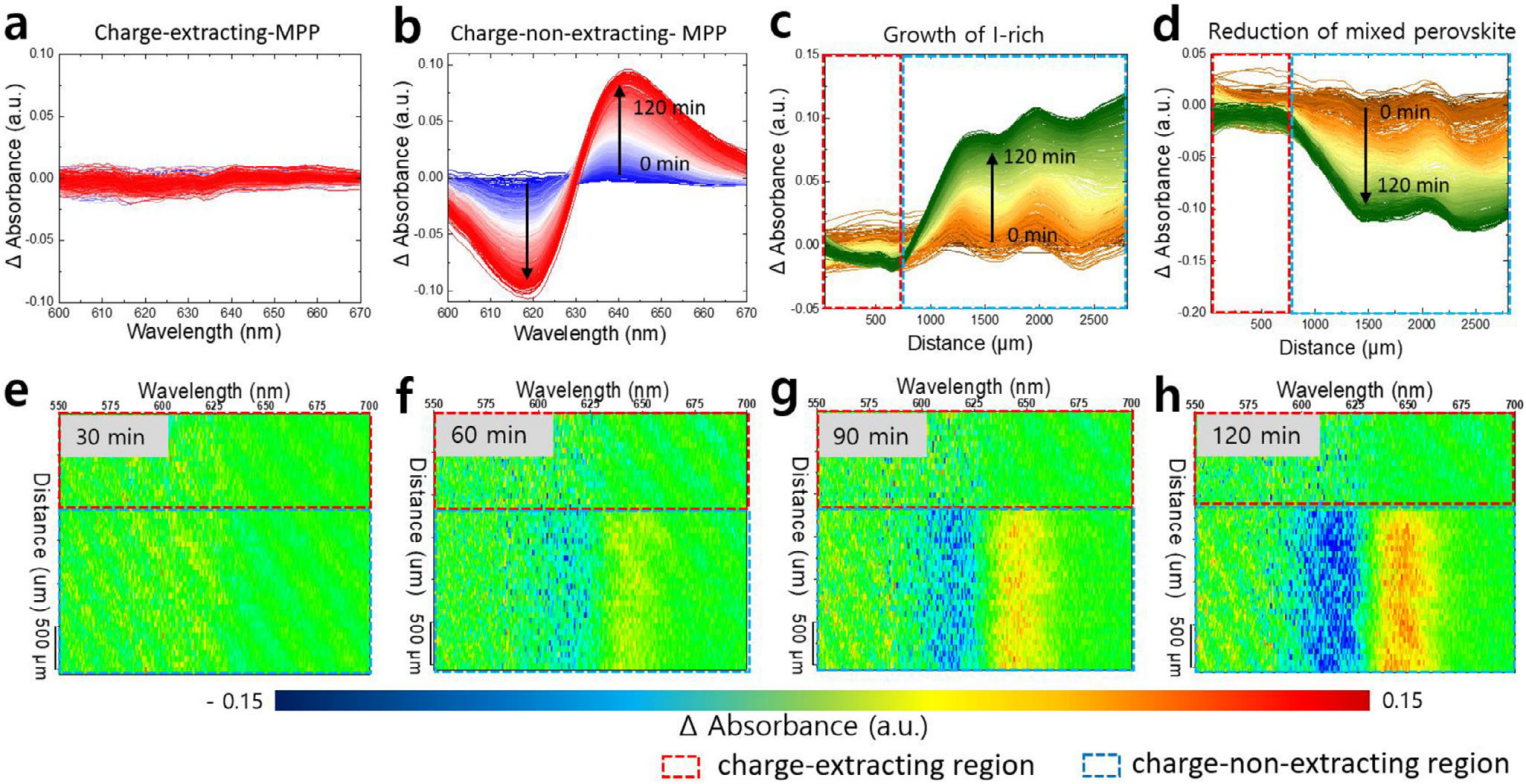
Time‐resolved ΔAbsorbance spectra under MPP conditions in the (a) charge‐extracting and (b) charge‐non‐extracting regions. Spatial line profiles at (c) 640 nm and (d) 620 nm showing growth of the iodine‐rich domains and depletion of the mixed‐halide phase, respectively. (e–h) 2D ΔAbsorbance maps recorded at (e) 30 min, (f) 60 min, (g) 90 min, and (h) 120 min under continuous MPP conditions, enabling visualization of the spatial and temporal evolutions of halide segregation.

To further investigate the spatial compositional changes associated with phase evolution under operational conditions, the differential absorbance (ΔAbsorbance) spectra were analyzed under MPP conditions based on the results presented in Figure 1 [29]. As shown in Figure 2a, the charge‐extracting region exhibits negligible changes in ΔAbsorbance across the full spectral window over 2 h of illumination. In contrast, Figure 2b shows that the charge‐non‐extracting region exhibits a progressive increase in ΔAbsorbance at ∼640 nm and a corresponding dip at ∼620 nm, indicating the formation of iodine‐rich domains and depletion of the original mixed‐halide composition, respectively. These spectral signatures are consistent with halide phase segregation previously reported for mixed‐halide perovskites under prolonged illumination. To quantify this spatial progression, the ΔAbsorbance line profiles were extracted at two representative wavelengths, namely 640 nm and 620 nm (Figure 2c,d, respectively). At 640 nm, the absorbance in the nonactive region steadily increased, indicating the growth of iodine‐rich phases. At 620 nm, the absorbance progressively decreased, indicating depletion of the original mixed‐halide composition. In both cases, the charge‐extracting region remained stable throughout the measurements, with ΔAbsorbance values close to zero.

Figure 2e–h shows the ΔAbsorbance heatmaps illustrating the spectral (x‐axis) and spatial (y‐axis) variations at 30 min, 60 min, 90 min, and 120 min, enabling the spatial visualization of simultaneous spectral changes across the device over time. Initially, both regions exhibit minimal spectral changes. By 60 min, phase segregation begins to emerge, becoming pronounced at 120 min, when the charge‐non‐extracting region exhibits a pronounced decrease in ΔAbsorbance at ∼620 nm and a pronounced increase in ΔAbsorbance at ∼640 nm, indicating a clear transition to a segregated phase [30]. In contrast, the charge‐extracting region exhibits negligible spectral changes, confirming its resistance to phase segregation. These observations support the hypothesis that segregation is kinetically suppressed by the continuous extraction of photocarriers. Photocarriers accumulate in the absence of a charge‐extracting structure, coinciding with the onset of phase separation even under typical MPP conditions, and evolving over time. Once iodine‐rich domains form, they locally reduce the bandgap and induce charge funneling, accelerating recombination and instability [5]. Figure S3 shows the absorbance heatmaps under different bias conditions, demonstrating that halide segregation is promoted under forward bias, but suppressed under reverse bias. These findings underscore the close relationship between charge extraction conditions and the kinetic evolution of the perovskite phase in terms of both the PV performance and the kinetic stability of the perovskite phase against ionic‐driven degradation [6].

### 2.2 Extension of the Spatial Origin to Module‐Scale Architectures

**FIGURE 3:**
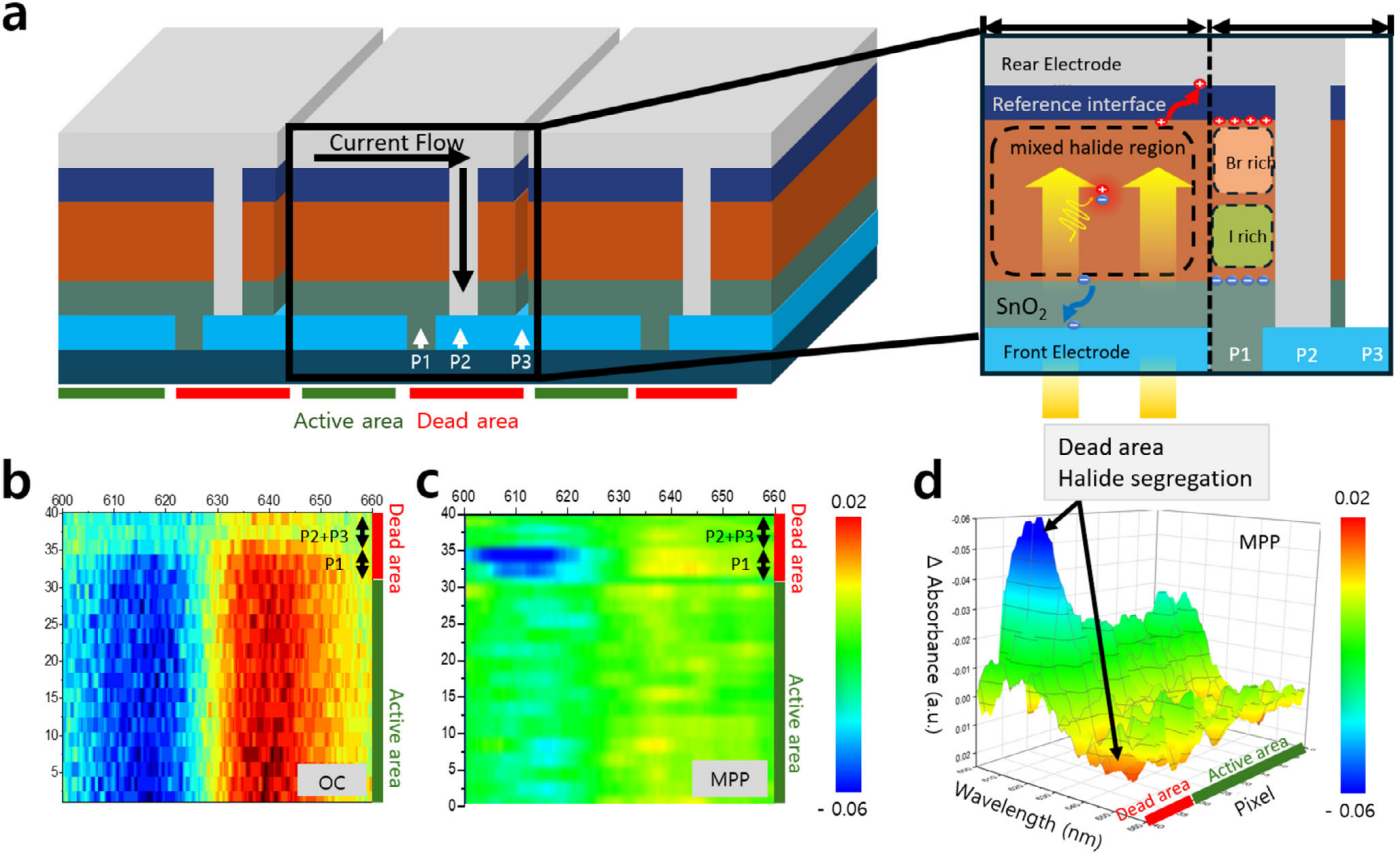
(a) Schematic representation of the perovskite module structure with laser‐scribed P1, P2, and P3 lines, illustrating the current flow and electrically inactive dead areas. 2D ΔAbsorbance maps of a reference perovskite module (b) OC and (c) MPPT conditions. (d) 3D ΔAbsorbance surface plot showing the wavelength‐ and position‐resolved absorbance changes even under MPP conditions, with pronounced spectral features localized in the dead region.

To evaluate the applicability of RTJAS at the module level, monolithic perovskite mini‐modules (series‐connected cells on a single substrate) were fabricated with patterned ITO top contacts and laser‐scribed P1–P3 lines (Figure 3a) [31]. This architecture includes charge‐non‐extracting interconnect zones (“dead areas”) between the active cells, which resemble practical module layouts. These regions can accumulate photoinduced carriers under bias and are prone to compositional instability under illumination. Figure 3a shows a cross‐sectional view near the scribe, highlighting halide redistribution in the dead zone, where mixed‐halide domains are observed, consistent with ion redistribution.

The reference CsPbI_2_Br module exhibited extensive halide segregation across most active areas during continuous illumination under OC conditions, as shown in the two‐dimensional (2D) ΔAbsorbance map (Figure 3b), revealing distinct iodide‐rich (∼650 nm) and bromide‐rich (∼590 nm) domains. In contrast, the areas near the P2+P3 scribes showed minimal changes due to the near‐complete absence of perovskite. Under MPP conditions, segregation was largely mitigated in the active regions, highlighting the stabilizing role of continuous carrier extraction. However, pronounced iodide‐rich features still appeared in the electrically inactive zones, particularly at the P1 edges and dead areas adjacent to the P2+P3 scribes. This localized segregation underscores the vulnerability of non‐extracting regions to photoinduced composition changes, even under operational conditions. The three‐dimensional (3D) spectral difference plot recorded under MPP conditions (Figure 3d) supports this result, showing partial halide segregation at the intercell gaps. These results indicate that efficient charge extraction in active regions does not necessarily extend to neighboring electrically inactive areas, where compositional instability can preferentially develop. Notably, such dead areas remain susceptible to phase separation even under MPP operation conditions, revealing a potential degradation pathway that is not captured by conventional device‐level measurements.

**FIGURE 4:**
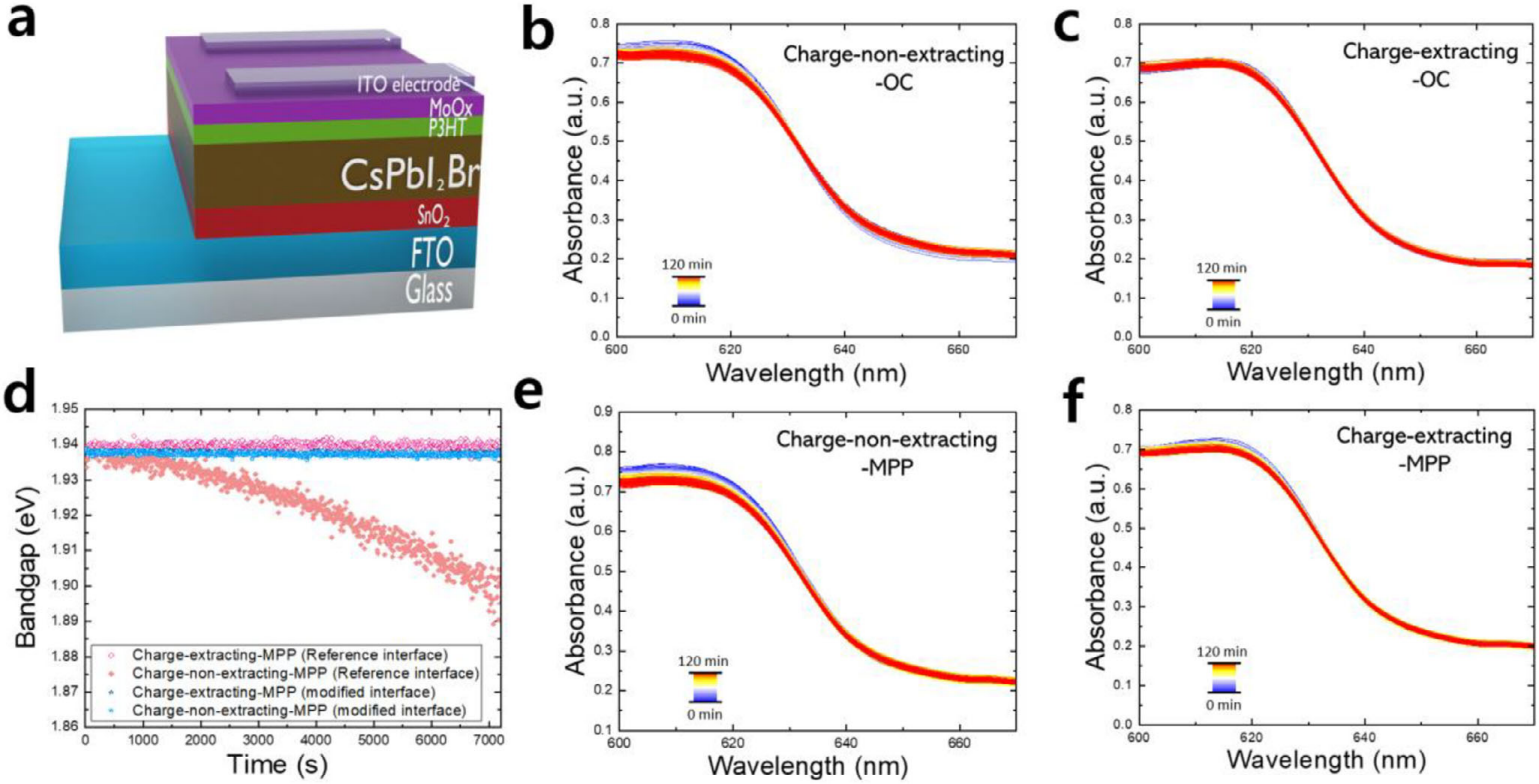
(a) Device architecture of a perovskite solar cell incorporating an organic interlayer (P3HT/MoO_x_) at the perovskite interface. (b,c,e,f) Time‐resolved absorbance spectra measured under identical operating conditions in the charge‐non‐extracting and charge‐extracting regions under (b,c) open‐circuit (OC) and (e,f) maximum power point (MPP) operation. (f) Time‐dependent evolution of the optical bandgap extracted under MPP conditions, showing spectrally stable behavior across both regions.

To examine how changes in interfacial configuration influence light‐induced phase evolution, the conventional inorganic hole transport layer was modified by introducing an organic interlayer structure consisting of P3HT/MoO_x_ (Figure 4a). In this configuration, the P3HT layer directly interfaces with the perovskite surface, while the ultrathin MoO_x_ layer (∼3 nm) is inserted between P3HT and the top ITO electrode. Owing to its limited thickness and comparatively low carrier mobility, MoO_x_ does not function as a bulk hole‐transport layer but rather serves as a hole‐selective contact. In addition, because the top ITO electrode was deposited by RF sputtering, the MoO_x_ layer acts as a protective interfacial buffer to mitigate sputter‐induced damage to the underlying P3HT layer. Therefore, the perovskite‐facing interface is defined by P3HT, whereas MoO_x_ primarily stabilizes the electrical contact during electrode deposition. This modification alters the interfacial chemistry while maintaining comparable device architecture [32, 33]. Using RTJAS, the time‐resolved absorption spectra of devices with the modified interfacial configuration were monitored under identical open‐circuit (OC) (Figure 4b,c) and maximum power point (MPP) (Figure 4e,f) conditions in both charge‐extracting and non‐extracting regions. Under these conditions, no pronounced red‐shift or long‐wavelength spectral evolution was detected within the measurement window, and the extracted bandgap values remained nearly constant over time (Figure 4d), even in regions without carrier extraction. This contrasting spectral behavior, observed under the same operating conditions used for the reference interface, suggests that the interfacial configuration influences the defect landscape associated with phase evolution. The absence of irreversible degradation (Figure S4) further confirms the ability of P3HT to preserve halide composition under operational conditions, establishing it as an effective interfacial strategy for wide‐bandgap perovskite devices. To elucidate the microscopic origin of this difference, defect spectroscopy and first‐principles calculations were performed.

### 2.3 Interfacial Defect Characteristics and Vacancy‐Related Trap States

**FIGURE 5:**
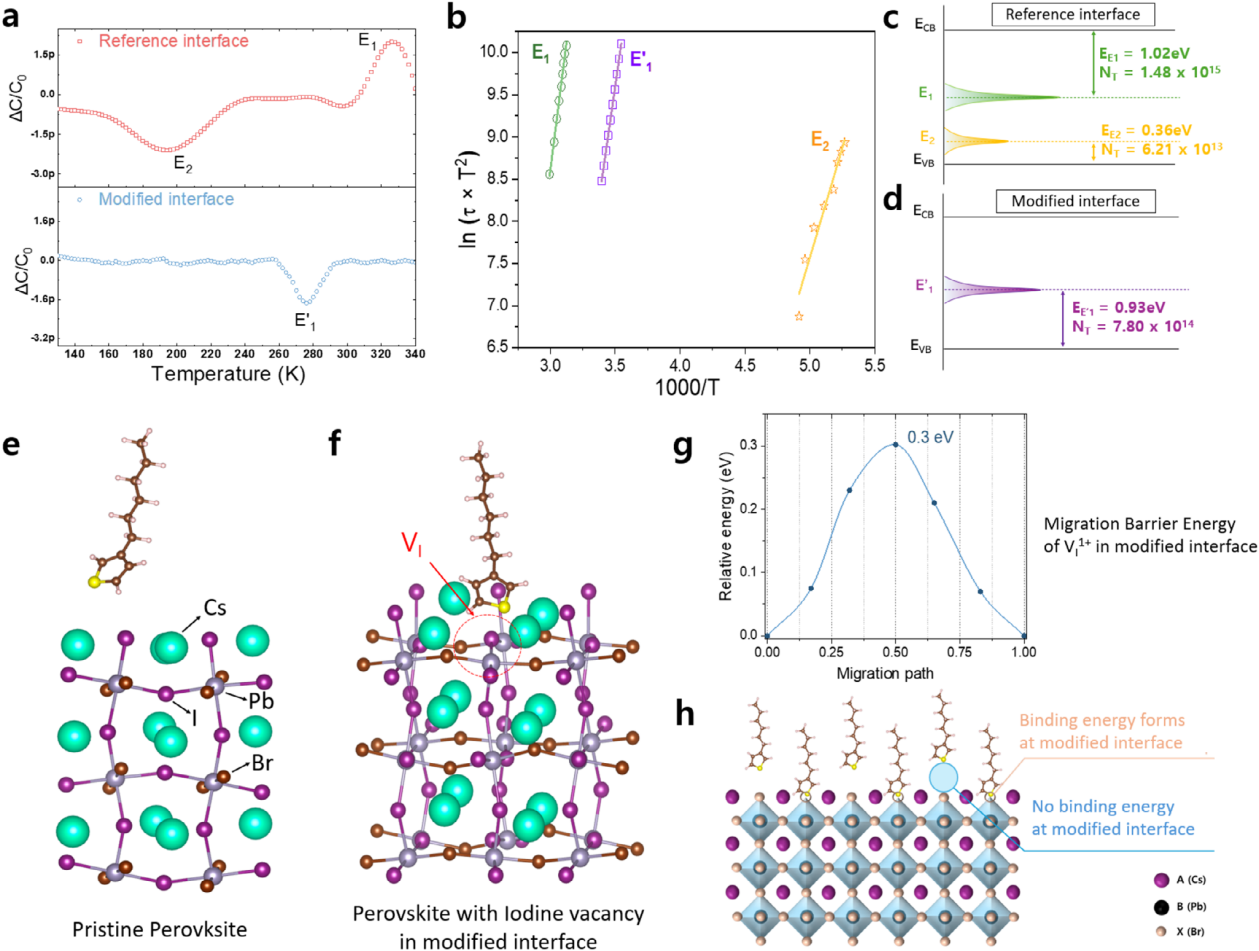
(a) DLTS spectra of the devices employing reference and modified interfaces, showing the E_1_, E_2_, and Eʹ_1_ trap states. (b) An Arrhenius plot is used to extract the trap activation energies. Energy‐level diagrams of the identified traps in the (c) modified interface and (d) reference interface‐based devices. Atomic models of (e) the pristine perovskite and (f) the perovskite with V_I_. (g) Calculated relative energy for V_I_^1+^ migration near the modified interface. (h) Interactions between the modified interface and V_I_, showing preferential interaction at V_I_.

DLTS was conducted to compare the transient charge responses associated with the trap states at the HTL perovskite interfaces in devices employing a reference interface and a modified interface incorporating an organic interlayer [34, 35]. As shown in Figure 5a, the reference‐interface device exhibits two distinct deep‐level traps, namely an electron trap (E_1_) and a hole trap (E_2_). In contrast, the modified‐interface device shows only a single hole‐related deep trap (E′_1_). Arrhenius analysis (Figure 5b) revealed the activation energies of these traps; E_1_ was located 1.02 eV below the conduction band, while E_2_ was located 0.36 eV above the valence band in the energy diagram of the reference‐interface device (Figure 5c). For the modified‐interface device, E′_1_ was located 0.93 eV above the valence band (Figure 5d). Trap states E_1_ and E′_1_ are energetically similar and are attributed to the presence of halide‐on‐lead antisite defects in their perovskite layers, in which a bromide ion occupies a Pb site (denoted as Br_Pb_). These defects introduce deep trap states within the bandgap [36, 37]. The Br_Pb_ antisite defects are structurally bound within the lattice, acting primarily as nonradiative recombination centers. Considering that E_1_ and E′_1_ are located near the mid‐gap of the perovskite, their behaviors as electron and hole traps are influenced by the band offset characteristics at the perovskite HTL interface. Specifically, because of differences in the Fermi level alignment at the interface (Figure S5), E_1_ behaves as an electron trap in the reference‐interface device, while E′_1_ acts as a hole trap in the modified‐interface device. Conversely, the E_2_ trap state in the reference‐interface device is typically associated with V_I_ (positively charged mobile ionic defects) in the perovskite layer [38, 39]. The modified‐interface device exhibits one deep trap (E′_1_) with a lower trap density and smaller capture cross‐sections (Table S1), resulting in slower trap‐assisted recombination. In contrast, the reference‐interface device contains two traps (E_1_ and E_2_) which accelerate nonradiative recombination, leading to greater degrees of charge loss and performance degradation [40]. Additionally, modified‐interface suppresses mobile V_I_ formation, thereby contributing to enhanced stability.

To elucidate the atomistic mechanism underlying V_I_ suppression at the modified‐interface, DFT calculations were performed. Figure 5e shows a pristine CsPbI_2_Br surface with fully occupied iodide sites, maintaining an undistorted Pb–I coordination and a defect‐free lattice [41]. Introducing V_I_ near the surface (Figure 5f) distorts the lattice and exposes under‐coordinated Pb^2^^+^ sites, which serve as initiation points for halide migration and ionic instability under bias [42]. As shown in Figure 5g, the calculated median migration energy barrier of V_I_^1+^ in the CsPbI_2_Br lattice is ∼0.3 eV, which closely matches the activation energy of E_2_ in reference‐interface devices.

Complementary DFT calculations performed for the multiple charge states (+1 and 0) and diffusion pathways of the iodine and Cs vacancies (Table S2) further supported the hypothesis that E_2_ originates from V_I_‐related trap states in reference‐interface devices. V_I_^1+^ species in the perovskite can migrate and accumulate at the HTL interface, contributing to unstable transient current responses. However, the absence of an E_2_‐like trap state in modified‐interface devices indicates that the modified‐interface suppresses both V_I_^1+^ formation and migration through interfacial interactions with the CsPbI_2_Br layer, thereby mitigating halide segregation [43]. When V_I_^1+^ is present, the sulfur atoms in the thiophene backbone coordinate with exposed Pb^2^^+^ species, forming weak donor–acceptor bonds that pin the vacancy [42]. This transforms mobile ionic defects into localized, electrically inert species.

### 2.4 Photovoltaic Performance and Stability Governed by Interfacial Defects

**FIGURE 6:**
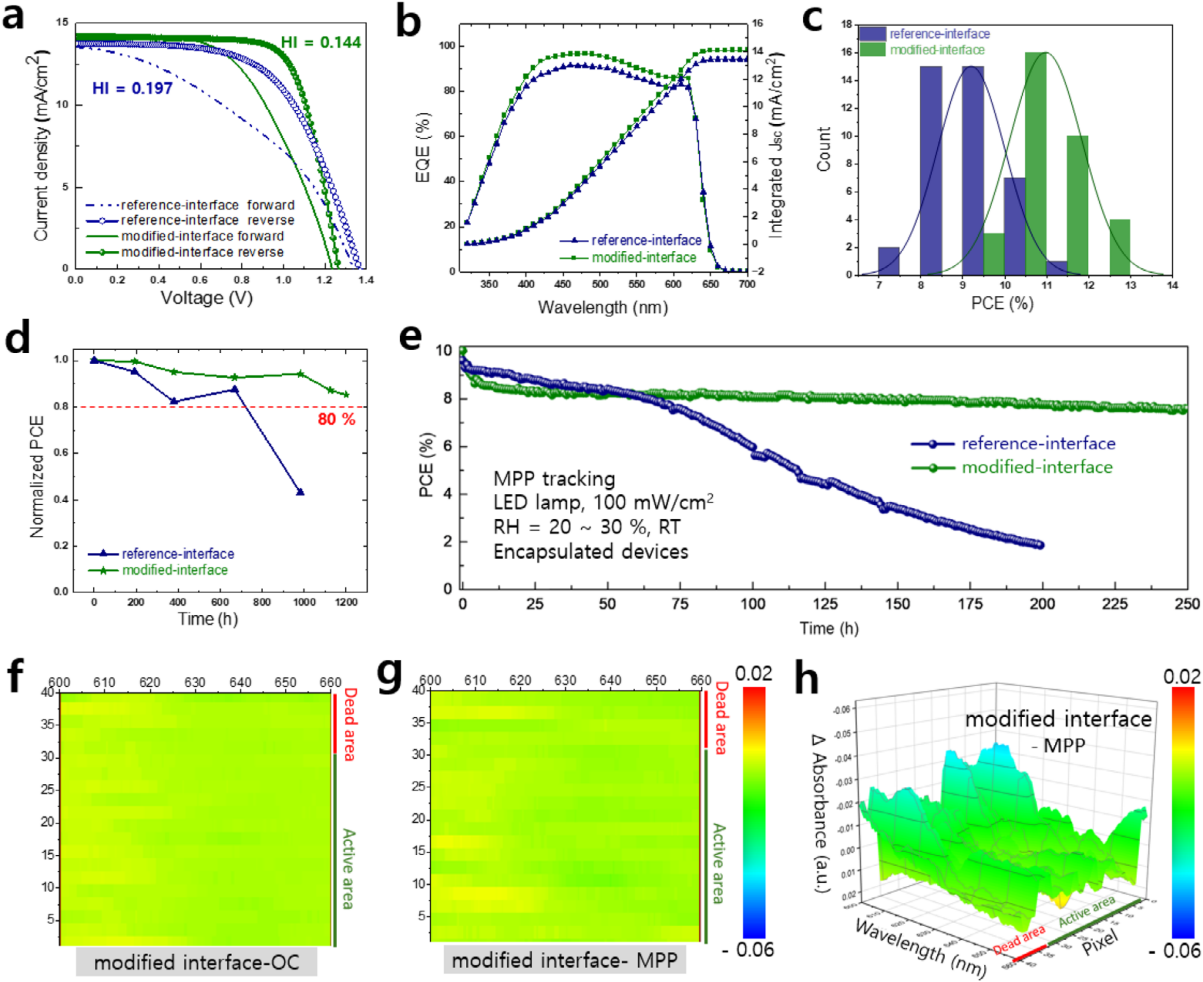
(a) *J–V* curves measured under AM 1.5G illumination, showing a lower hysteresis index (HI) for the modified‐interface device than for the reference‐interface device. (b) EQE spectra and integrated J_SC_ for both devices. (c) PCE distribution. (d) Long‐term stability under ambient dark storage conditions, showing the normalized PCE over 1200 h. (e) Operational stability under continuous MPP conditions at 100 mW cm^−2^, demonstrating enhanced operational durability for the modified‐interface device. 2D ΔAbsorbance maps of a module incorporating the modified interface under (f) OC and (g) MPP conditions. (h) 3D ΔAbsorbance surface plot for the module with the modified interface under MPP conditions.

Figure 6 and Figure S6 present a comparison of photovoltaic performance and stability for CsPbI_2_Br devices employing reference versus modified interfaces. The modified‐interface device exhibits a higher fill factor (FF) and a reduced hysteresis index (HI: 0.144 vs. 0.197), indicating suppressed ionic defects and more stable charge extraction (Figure 6a) [44]. The external quantum efficiency (EQE) spectrum (Figure 6b) for the modified‐interface device maintains a strong spectral response across the visible range, which is comparable to or slightly higher than that of the reference‐interface device. The integrated short‐circuit current density (J_sc_) closely matches the values obtained from the *J–V* measurements, confirming the accuracy of the optical‐to‐electrical conversion and the minimal photocurrent losses in both architectures. The enhanced PCE of the modified‐interface device therefore stems from more efficient carrier extraction and transport, as well as from reduced interfacial recombination. The PCE histogram (Figure 6c) reveals a higher average efficiency for the modified‐interface device (∼11.5%) compared to that for the reference‐interface device (∼9%). This was attributed to effective surface defect passivation, which improves the overall photovoltaic performance. Thus, in addition to the initial efficiency, the interfacial defect passivation also significantly affects the device stability. Stability testing under dark ambient storage conditions (Figure 6d) demonstrated a PCE retention of >90% over 1000 h for the modified‐interface device, while that of the reference‐interface device dropped below 80% after 100 h. Under continuous MPP illumination (100 mW cm^−2^ LED, 20–30% RH), the reference‐interface device retained only ∼20% of its initial PCE after 200 h, while the modified‐interface device retained ∼80% (Figure 6e). Notably, the modified‐interface device exhibits a modest initial PCE decrease under MPP tracking, which can be attributed to transient interfacial equilibration under illumination and bias. Such early‐stage efficiency drift is associated with ionic redistribution and field screening [11]. Following this equilibration period, the device displays a significantly slower decay rate, resulting in superior long‐term operational stability compared to the reference‐interface device. These results highlight the role of interfacial defect passivation in mitigating photoinduced degradation mechanisms, such as halide ion migration and trap formation, which are more pronounced in reference‐interface devices [45].

It is noteworthy that, as shown in Figure S5, the reference‐interface exhibits a smaller valence band offset compared to the modified‐interface, suggesting more favorable energetic alignment for hole extraction from a purely electronic standpoint. Nevertheless, the reference‐interface devices display significantly poorer operational stability. This comparison indicates that favorable band alignment alone does not ensure long‐term durability. Because degradation in these materials is closely associated with ionic defect formation and halide migration under charge accumulation, the chemical characteristics of the interface become critically important. In the modified‐interface devices, the organic interlayer directly interacts with the perovskite surface and suppresses iodine vacancy migration, as supported by the DLTS and DFT analyses. Therefore, even with a slightly larger valence band offset, the modified‐interface devices provide superior phase stability.

The module incorporating the modified interface exhibits no pronounced spectral signatures of phase separation in either the active or dead regions. No new absorption peaks or spectral shifts were observed in the dead areas during prolonged illumination under OC conditions. As shown in Figure 6f, the entire mapped region remains spectrally uniform, indicating retention of a single‐phase mixed‐halide composition without iodide‐ or bromide‐rich domains. Similarly, under MPP conditions (Figure 6g), the perovskite film maintained a stable composition. The 3D difference map (Figure 6h) is essentially flat, lacking pronounced features in either region. This absence of segregation indicates that interfacial defect passivation by the modified interface is effective in both the biased areas and in electrically inactive zones. The uniform iodine and bromine distribution, even in regions where photogenerated carriers accumulate, suggests that the modified interfacial configuration alters charge accumulation and ion migration behavior that typically drive ion migration and phase separation.

## 3 Conclusion

This study presents an integrated operando and defect‐level framework to understand and address phase instability in wide‐bandgap mixed‐halide perovskite photovoltaics. Using real‐time current density–voltage absorption spectroscopy (RTJAS) with a transparent ITO top contact, light‐induced phase evolution was directly visualized across charge‐extracting and non‐extracting regions under operational conditions. This spatially resolved approach reveals that phase instability preferentially initiates in regions with locally insufficient carrier extraction, highlighting the importance of interfacial defect activity in governing phase behavior.

Importantly, this spatial origin is preserved when extending from device‐scale measurements to monolithic module architectures, where electrically inactive dead areas act as preferential sites for compositional instability. Correlating these operando observations with defect spectroscopy and first‐principles calculations indicates that differences in interfacial defect landscapes critically influence vacancy‐related processes and phase evolution.

Devices and modules incorporating a modified interfacial configuration exhibit improved photovoltaic performance and enhanced resistance to photoinduced degradation without altering the bulk perovskite composition. These findings underscore interfacial defect regulation as a viable and scalable strategy for mitigating phase instability and provide practical design guidelines for achieving durable perovskite photovoltaics from device to module scale. Importantly, these findings indicate that phase instability extends beyond a bulk compositional phenomenon and is spatially governed by local electrical boundary conditions and charge extraction asymmetry. Recognizing this device‐level contribution may provide a complementary pathway for improving the stability of scalable mixed‐halide perovskite devices.

## 4 Experimental Section

### 4.1 Materials

Tin(II) chloride dihydrate (SnCl∙2H_2_O), urea, thioglycolic acid, methylammonium chloride (MACl), cesium iodide (CsI), sodium formate, lead acetate trihydrate (PbAc∙3H_2_O), molybdenum(VI) oxide (MoO_x_), diphenyl ether, nickel(II) acetylacetonate, oleylamine, 1‐butanol, chlorobenzene (CB), chloroform, dimethyl sulfoxide (DMSO), and N,N‐dimethylformamide (DMF) were purchased from Sigma Aldrich. Lead bromide (PbBr_2_) and lead iodide (PbI_2_) were purchased from TCI. P3HT was purchased from Rieke Metals. All materials were used without further purification or recrystallization.

### 4.2 Synthesis of the NiO_x_ NPs

Nickel(II) acetylacetonate (0.5138 g) was dissolved in a mixed solution of oleylamine (20 mL) and toluene (20 mL) under magnetic stirring for a few minutes to form a dark blue solution. This solution was heated in an oven at 190°C for 24 h. After cooling to room temperature, a clear green solution was obtained. Subsequently, acetone was added to the solution, and the solution was centrifuged to isolate the NiO_x_ nanoparticles (NPs). The NiO_x_ NPs were thoroughly dried and dispersed in a mixed solvent (chloroform/1‐butanol (4:1 v/v)) to give a concentration of 36 mg/mL.

### 4.3 Device Fabrication

Fluorine‐doped tin oxide (FTO)‐coated substrates (Asahi glass) were etched and washed with deionized water, acetone, and isopropanol for 15 min each. To perform the chemical bath deposition (CBD) of SnO_2_, a solution was prepared by dissolving urea (5 g), SnCl∙2H_2_O (1.096 g), thioglycolic acid (100 µL), and HCl (5 mL) in deionized water (400 mL). The resulting solution was stirred in ambient air for 1 h. The cleaned substrates were then immersed in the CBD solution at 70°C for 12 h, followed by washing for 5 min with deionized water and annealing at 150°C for 1 h. The resulting SnO_2_ films on the FTO substrates were subjected to ultraviolet–ozone treatment for 30 min. A 1.3 m CsPbI_2_Br perovskite precursor solution was prepared by dissolving CsI (1.3 m), PbI_2_ (0.68 m), PbBr_2_ (0.65 m), PbAc∙3H_2_O (2 mol%), sodium formate (2 mol%), and MACl (0.025 g) in a mixed solvent (1 mL) composed of DMF and DMSO (2:3, v/v). The precursor solution was subsequently spin‐coated on the FTO/SnO_2_ substrate using a two‐step program, namely 500 rpm for 3 s and 3500 rpm for 80 s. The films were then annealed at 185°C for 10 min. Deposition of the CsPbI_2_Br film was conducted at a relative humidity of <20%. For the NiO_x_‐based device, a solution was prepared by mixing chlorobenzene (1:1, v/v) with the previously synthesized NiO_x_ NPs. This mixture was spin‐coated at 2000 rpm for 30 s and 5000 rpm for 20 s, followed by annealing at 120°C for 10 min. For the P3HT‐based device, a solution was prepared by dissolving 10 mg/mL of P3HT in CB, followed by the addition of diphenyl ether (30 µL). This solution was spin‐coated at 3000 rpm for 30 s. Subsequently, a 3 nm MoO_x_ layer was deposited via thermal evaporation. Finally, the ITO electrode was sputter‐coated onto the substrate for over 20 min using a radio frequency power of 200 W, while maintaining the substrate temperature at 50°C.

### 4.4 Modules

The perovskite solar modules were fabricated in a monolithic configuration using laser scribing with a nanosecond‐pulsed 355‐nm Nd:YAG system (EO Technics). P1–P3 scribing was performed at repetition rates of 130–200 kHz and diode currents of 4.6–5.8 A, optimized for each patterning step. The resulting line widths for P1, P2, and P3 were approximately 15, 44, and 22 µm, respectively, yielding a total dead width of ∼240 µm.

### 4.5 Device Characterization

The *J–V* curves were measured using a Class A solar simulator (Newport, 91195A) equipped with a source meter (Keithley 2420) under AM 1.5G illumination, which was calibrated using an NREL‐certified Si reference cell. For characterization of the *J–V* curve, the step voltage and scan speed were fixed at 10 mV and 100 mV s^−1^, respectively. For high‐performance devices, an antireflection film was applied to the rear side (glass side) of the device. During measurement of the photovoltaic performance, all devices were covered with a metal mask featuring a circular aperture of 0.096 cm^2^. The EQE was measured using a QUANTX‐300 QE system with a 100 W xenon lamp and a 130 mm focal length monochromator with dual gratings. The operational stability was assessed by tracking the MPP under 100 mW cm^−2^ continuous illumination (LED solar simulator, LSH‐7320, Newport) at room temperature under air.

RTJAS was performed using a custom‐built optical platform [10]. The device was illuminated with collimated broadband light delivered through a vertical slit aperture and focused onto the sample using plano‐convex lenses. The collimated beam had a diameter of 48 mm at the sample plane, while the RTJAS‐probed region (3 mm) was centrally positioned within this area. Spatial and spectral homogeneity were confirmed from the broadband background image, where spectra extracted across multiple spatial positions exhibited intensity variations within 2% over the measured wavelength range (Figure S7). A transparent ITO top contact enabled localized charge extraction, while maintaining optical access to both the charge‐extracting and non‐extracting regions. Transmitted light was collected using an off‐axis parabolic mirror, dispersed by a diffraction grating, and imaged onto a CMOS camera. The spatial resolution of the RTJAS system is governed by the optical magnification and CMOS pixel size, yielding an effective spatial resolution of approximately 62 µm in the current configuration. The detector operated at 155 frames per second, corresponding to a temporal resolution of ∼6.5 ms per frame. This configuration provided two‐dimensional spectral data, wherein the horizontal axis corresponded to the wavelength‐dependent absorbance, and the vertical axis resolved spatial differences along the lateral device plane. For baseline correction, reference spectra were acquired in the absence of a sample. Simultaneous *J–V* measurements were carried out by applying an external bias (0–1.25 V, 0.01 V steps) using micromanipulated probes contacting the front and back electrodes. This setup enabled the operando, spatiotemporally resolved monitoring of the optical and electrical changes under both OC and MPPT conditions.

DLTS was conducted using a cryogenic probe station (Lakeshore TTPX) equipped with an impedance analyzer (Zurich Instruments MFIA). To enhance the signal reliability, temperature‐dependent scans were performed from 100 K to 395 K with a 1 K increment, averaging 10 measurements per step. To assess the doping density and depletion width, the capacitance–voltage (C–V) profiles were recorded at 10 kHz. A voltage pulse ranging from −0.5 V to 0 V was applied for DLTS, along with a pulse width of 1 s and a period of 5 s. DLTS detects deep‐level defects by monitoring capacitance transients after a voltage pulse, which perturbs the steady‐state depletion region; the subsequent thermal emission of trapped carriers enables the extraction of defect energy levels, capture cross‐sections, and concentrations, providing valuable insights into carrier recombination processes in semiconductors.

First‐principles DFT calculations were performed to determine the migration barrier energy of the native defects and the adsorption energy of a P3HT molecule on the perovskite surface. The electron–ion interactions were described using the projected augmented‐wave method [46], as implemented in the Vienna Ab initio Simulation Package [47]. The Perdew–Burke–Ernzerhof exchange‐correlation function was applied [48]. A plane‐wave energy cutoff of 297.3 eV was used, and the structure was relaxed until the residual forces were <0.03 eV/Å. The climbing nudged elastic band method was employed to calculate the migration barrier energy [49].

## Conflicts of Interest

The authors declare no conflicts of interest.

## Supporting information




**Supporting File**: advs74909‐sup‐0001‐SuppMat.docx.

## Data Availability

All the relevant data supporting this study are available from the corresponding author on reasonable request.
