# Supplementary material for: Engineered Interfacial Control for Suppression of Phase Instability: Operando Visualization from Device to Module Scale
Source: Adv Sci (Weinh). 2026 Apr 2;13(31):e74909. doi: 10.1002/advs.74909 (PMC13588056; doi:10.1002/advs.74909)
Supplement: Supplementary file 1 [file ADVS-13-e74909-s001.docx]

Engineered Interfacial Control for Suppression of Phase Instability:
Operando Visualization from Device to Module Scale

Hangyeol Choi,^a^† Soo Woong Jeon,^b^† Wookyung Jeon,^a^ You‐Hyun Seo,^c^ Gill Sang Han,^c^ Myunghun Shin,^d^ Ji-Sang Park_,_*^e^ Jun Hong Noh,*^b^ and Yohan Yoon*^a^

H. Choi, W. Jeon, Y. Yoon

Korea Aerospace University, Department of Materials Engineering, Goyang 10540, Republic of Korea.

Email: yyoon@kau.ac.kr

S. W. Jeon, J. H. Noh

School of Civil, Environmental and Architectural Engineering, Korea University, Seoul 02841, Republic of Korea

Email: junhnoh@korea.ac.kr

Y.-H. Seo, G. S. Han

Division of Advanced Materials, Korea Research Institute of Chemical Technology (KRICT), Daejeon 34114, Republic of Korea

M. Shin

School of Electronics and Information Engineering, Korea Aerospace University, Goyang 10540, Republic of Korea

J.-S. Park

SKKU Advanced Institute of Nano Technology, Sungkyunkwan University, Suwon 16419, Republic of Korea

Email: jisangpark@skku.edu

† These authors contributed equally to this work.

Under continuous illumination, the mixed-halide perovskite exhibits systematic spectral evolution across the absorption window (Figure S1). As discussed in the main text, the most prominent feature is the progressive redshift of the absorption onset from 620 nm toward longer wavelengths, consistent with the formation of iodine-rich domains possessing reduced bandgaps. This redshift develops gradually over the course of illumination and reflects compositional redistribution driven by charge accumulation.

In addition to the redshifted absorption edge, subtle but reproducible changes are observed around 590 nm. Rather than indicating a simple bandgap shift toward a bromide-rich end member, this feature reflects redistribution of phase fractions within the mixed-halide absorber. The concurrent evolution at both the long-wavelength (iodine-rich) and shorter-wavelength (590 nm) regions indicates bidirectional compositional segregation into iodine-enriched and bromine-enriched domains. The presence of these coordinated spectral changes supports the interpretation that the RTJAS signal originates from dynamic halide redistribution rather than from a uniform bandgap drift or purely defect-induced absorption tailing.

**Figure S1.** Time-dependent absorption spectra of the mixed-halide perovskite under continuous illumination, showing progressive redshift of the absorption onset associated with iodine-rich domain formation and concurrent spectral evolution near ~590 nm consistent with bromine-enriched regions during segregation.

The effects of electrical operating conditions of a device on halide phase stability were assessed by cycling the device between short-circuit (SC) and open-circuit (OC) states, while monitoring the bandgap evolution using real-time spectroscopy (Figure S2). In the OC state, the absence of charge extraction facilitated ion accumulation and promoted halide segregation, as evidenced by a rapid red-shift in the bandgap energy. In contrast, operation in the SC state maintained quasi-equilibrium conditions, suppressing segregation dynamics or enabling partial recovery. OC states can realistically occur in photovoltaic power systems during inverter faults, grid disconnection, or output curtailment events, where the generated current cannot be delivered to the load. Under these conditions, prolonged exposure to OC bias can cause ionic redistribution and irreversible phase degradation, particularly in halide perovskite devices. These findings indicate that the periodic interruption of carrier extraction, such as during transient disconnections in real-world applications, can critically destabilize halide distribution, even within the charge-extracting region.

**Figure S2.** Continuous optical bandgap evolution in the charge-extracting region of a NiOₓ-based device under alternating charge extraction conditions.

Three-dimensional ∆Absorbance maps of a CsPbI_2_Br device were recorded in the charge-extracting region under varied applied biases (from −1 V to +1 V in 0.5 V increments). The spectral peak at ~620–640 nm corresponds to the mixed-halide phase. A decrease in the absorbance intensity indicates progressive halide segregation and the formation of an iodine-rich domain. A significant decrease in ∆Absorbance was observed with an increasing forward bias, indicating that electric field-induced charge accumulation accelerates phase separation. In contrast, under a reverse bias (−1 V), segregation was strongly suppressed, highlighting the effects of the field polarity and the carrier distribution on the halide migration modulation.

**Figure S3.** Voltage-dependent spatial absorbance mapping of the field-driven phase instability.

As shown in Figure S4a, OC conditions significantly accelerate halide segregation in the NiOₓ-based CsPbI_2_Br device, particularly in the charge-non-extracting regions. The resulting absorbance loss is only partially reversible, even after extended dark recovery (>20 h), thereby indicating a transition from reversible ionic redistribution to irreversible phase decomposition. This irreversible degradation under repeated OC stress highlights the critical vulnerability of reference interfaces under real-world operating conditions. In contrast, Figure S4b shows that the modified-interface device exhibits negligible changes in absorbance across all cycles, regardless of charge extraction. The suppression of halide segregation appears intrinsic and robust, indicating that modified-interface not only delays degradation, but also prevents phase instability via effective interfacial passivation. These findings emphasize the importance of interface design in determining the reversibility of halide-related degradation and ensuring long-term device stability under fluctuating bias conditions.

**Figure S4.** Comparative reversibility of halide segregation under operational, open-circuit, and recovery conditions.

Figure S5 shows the ultraviolet photoelectron spectra (left: secondary electron cutoff, right: valence region) and the corresponding energy level diagrams for the perovskite, NiOₓ, P3HT, and MoOₓ films. It can be seen that the reference-interface exhibits a poor alignment with the perovskite valence band, introducing energetically deep states that can trap carriers. In contrast, modified-reference exhibits a favorable energy alignment and smaller interfacial band offsets, thereby suppressing nonradiative recombination and defect formation.

**Figure S5.** Ultraviolet photoelectron spectra and energy level alignment of the interfacial layers.

The normalized power conversion efficiencies (PCEs) of the CsPbI_2_Br devices based on the reference-interface and modified-interface were measured using real-time current density–voltage absorption spectroscopy (RTJAS). The modified-interface devices retained >80% of their initial PCE over 7000 s, whereas the reference-interface devices retained <60%. These results highlight the role of interfacial passivation in mitigating defect-induced degradation during operation.

**Figure S6.** Comparative operational stabilities of the reference-interface and modified-interface based devices monitored using RTJAS.

The device was illuminated using a broadband xenon arc lamp (Newport 66921, 450–1000 W) coupled with a dual plano-convex F/1 lens system, producing a collimated beam of approximately 48 mm in diameter at the sample plane. The RTJAS-probed region (charge-extracting and non-extracting areas) spanned ~3 mm and was centrally positioned within the beam to ensure uniform illumination. Spatial and spectral homogeneity were verified from the broadband background image (Fig. 1g), where spectra extracted across 19 spatial positions exhibited intensity variations within ~2% over the measured wavelength range.

**Figure S7.** Spectral uniformity of the broadband illumination. Spectra extracted from 19 spatial positions (vertical axis in Fig. 1g) show intensity variations within ~2% across the measured wavelength range, confirming high spatial and spectral homogeneity.

.

**Table S1.** Trap parameters, including the activation energies, trap densities, capture cross-sections, and defect identifications.

| Sample | Activation energy (eV) | Trap density  (cm^−3^) | Capture cross-section (cm^2^) | Defect identification |
| --- | --- | --- | --- | --- |
| Modified interface | 0.93 | 7.80 × 10^14^ | 5.96 × 10^−15^ | Br_pb_ (antisite) |
| Reference interface | 0.36 | 6.21 × 10^13^ | 8.43 × 10^−18^ | V_I_ |
|  | 1.02 | 1.48 × 10^15^ | 6.20 × 10^−15^ | Br_pb_ (antisite) |

To investigate the migration characteristics of intrinsic point defects in CsPbI_2_Br perovskites, density functional theory (DFT) calculations were performed for the iodine and cesium vacancies in both the neutral (0) and positively charged states (+1) across various migration pathways.

**Table S2.** DFT-calculated migration pathways and charged states (0 and +1) of the iodine and cesium vacancies in CsPbI_2_Br, illustrating an intrinsic defect mobility.

| Defect | Charged state | Pathway | Barrier energy (eV) |
| --- | --- | --- | --- |
| Iodine vacancy | 1+ | Path 1 | 0.3025 |
|  | 1+ | Path 2 | 0.2982 |
|  | 0 | Path 1 | 0.362 |
|  | 0 | Path 2 | 0.3903 |
| Cs vacancy | 1− | Path 1 | 0.6145 |
|  | 1− | Path 2 | 0.7321 |
|  | 0 | Path 1 | 0.6148 |
|  | 0 | Path 2 | 0.7184 |
